# What are the roles of antibodies versus a durable, high quality T-cell response in protective immunity against SARS-CoV-2?

**DOI:** 10.1016/j.jvacx.2020.100076

**Published:** 2020-08-28

**Authors:** Marc Hellerstein

**Affiliations:** University of California at Berkeley, United States; San Francisco General Hospital, University of California at San Francisco, United States

**Keywords:** SARS-CoV-2, SARS, COVID-19, Protective immunity, T-cells, CD8 T-cells, Antibodies, T cell lifespan, Durable immunity, Antibody-dependent enhancement, T-cell epitopes, Vaccines, Yellow Fever Vaccine

## Abstract

The first SARS-CoV-2 vaccine(s) will likely be licensed based on neutralizing antibodies in Phase 2 trials, but there are significant concerns about using antibody response in coronavirus infections as a sole metric of protective immunity. Antibody response is often a poor marker of prior coronavirus infection, particularly in mild infections, and is shorter-lived than virus-reactive T-cells; strong antibody response correlates with more severe clinical disease while T-cell response is correlated with less severe disease; and antibody-dependent enhancement of pathology and clinical severity has been described. Indeed, it is unclear whether antibody production is protective or pathogenic in coronavirus infections. Early data with SARS-CoV-2 support these findings. Data from coronavirus infections in animals and humans emphasize the generation of a high-quality T cell response in protective immunity. Yellow Fever and smallpox vaccines are excellent benchmarks for primary immune response to viral vaccination and induce long-lived virus-reactive CD8 T-cells, which are present and measurable within 1–4 months of vaccination. Progress in laboratory markers for SARS-CoV2 has been made with identification of epitopes on CD4 and CD8 T-cells in convalescent blood. These are much less dominated by spike protein than in previous coronavirus infections. Although most vaccine candidates are focusing on spike protein as antigen, natural infection by SARS-CoV-2 induces broad epitope coverage, cross-reactive with other betacoronviruses. It will be important to understand the relation between breadth, functionality and durability of T-cell responses and resulting protective immunity. It would be a public health and general trust-in-medicine nightmare - including a boost to anti-vaccine forces - if immune protection wears off or new disease patterns develop among the immunized. Data correlating clinical outcomes with laboratory markers of cell-mediated immunity, not only with antibody response, after SARS-CoV-2 natural infection and vaccines may prove critically valuable if protective immunity fades or if new patterns of disease emerge.

## 1 Introduction

The most definitive solution to the current world-wide public health and economic crisis will be an effective vaccine against SARS-CoV-2 (COVID-19). But due to the urgency of this moment, the first SARS-CoV-2 vaccine(s) will likely be licensed based on laboratory evidence of neutralizing antibodies in earlier (Phase 2) trials, prior to Phase 3 efficacy and safety trials.

## 2 Immunologic concerns about using antibody response as a sole metric of protective immunity in coronavirus infections

Both humoral immunity and cell mediated immunity, particularly from CD8 T-cells, play key roles in vaccine-induced protective immunity against intracellular infections like viruses [1]. For coronavirus infections including SARS-Cov-2, the literature is striking on this topic and raises important concerns.

1. Antibody response is not a great marker of coronavirus infection. T-cell responses have been better markers than antibody responses after natural coronavirus infection [2], [3], [4], [5], [6], [7], [8], [9], [10]. In severe acute respiratory syndrome (SARS), only 50% of survivors had detectable antibodies at 3 years and none had antibodies or B-cell responses to SARS-CoV-1 at 6 years, while virus-specific T-cells remain at 6–17 years [8], [9], [2], [3], [4]. Antibody response in Middle East respiratory syndrome (MERS) is low or absent in mild disease [5]. Experimental infection with a common cold coronavirus in humans resulted in antibodies that died away within 1 year [6]. Early data in SARS-CoV-2 infection suggest short-lived, less robust or absent antibody response in mild clinical disease, with 40% of asymptomatic patients being seronegative for anti-spike IgG ~12 weeks after virologic diagnosis and a 70% mean reduction from initial IgG levels [7], [10].

2. Strong antibody response correlates with more severe clinical disease while strong T-cell response is correlated with less severe disease. MERS survivors with higher antibody levels had experienced longer ICU stays and required more ventilator support compared to subjects with no detectable antibodies [11], while higher virus-specific T-cell counts were observed with no detectable antibodies in recovered patients who had less severe disease. The authors [11] proposed that T-cells clear virus rapidly, which reduces disease severity, exposure to virus and the strength of antibody response. Higher IgG levels against spike protein during acute infection were observed in SARS patients who subsequently died, associated with worse clinical lung injury and pro-inflammatory macrophages, compared to SARS patients who went on to recover [12] In COVID-19 patients, total T cells counts are markedly lower in most patients compared to healthy controls and low CD8 T-cell counts (<165/mcl) are a predictor of higher risk for death [13], [14]. Antibody response is higher in severe disease than in milder disease [15] and an abrupt elimination of virus is not observed after the appearance of antibodies [16].

3. Antibodies can worsen disease (antibody-dependent enhancement) in coronavirus infection in animals and possibly humans. Feline infectious peritonitis is a coronavirus disease. Severity is worsened by vaccination or passive immunization with serum from cats containing high antibody titers prior to viral infection [17]. SARS-CoV-1 virus causes hepatitis in ferrets only in previously vaccinated animals [18]. In macaques, administering immunoglobulin against spike protein worsened subsequent SARS-CoV-1-induced lung damage, induced inflammatory cytokines and reduced wound healing [12]. These findings parallel dengue hemorrhagic fever in humans, where initial infection and antibody response followed by a second infectious episode is required for serious hemorrhagic disease. Subneutralizing antibodies can promote viral entry into cells, including entry into and activation of macrophages and can occur through low-affinity antibodies, cross-reactive antibodies to different strains, or suboptimal titers of neutralizing antibodies. Vaccine trials for feline infectious peritonitis and dengue had to be halted because of disease enhancement. It has to be considered that antibodies alone might worsen coronavirus disease severity. Emerging data in COVID-19 patients support this concern. High serum IgG levels against SARS-CoV-2 are associated with more serious disease [19], [20]. As Cao wrote [19], “significant antibody production is observed; however, whether this is protective or pathogenic remains to be determined.”

T-cells might also amplify tissue damage in lung and heart in established coronavirus infection or after vaccination, due to cytokine excess or an eosinophilic proinflammatory Th2 response of CD4 T-cells [21]. Although eosinophilic Th2 lung damage was reported in mice from viral challenge after a SARS-CoV-1 vaccine [22], data in COVID-19 patients do not show a Th2 cytokine profile [23], [24]. In mouse models of SARS, virus-specific T-cells are necessary and sufficient for protection against disease [25], [26], [27].

Several authors have come to the conclusion that a coronavirus vaccine should optimally induce virus-specific T-cells, not just antibodies. Zhao [11] wrote, “future vaccines against emerging coronaviruses should emphasize the generation of a memory CD8 T cell response for optimal protection” and Liu [12] concluded “in addition to a strong anti-SARS-CoV antibody response, an optimal memory CD8 T cell response will be an important goal in vaccine design”.

## 3 Prevalence and specificity of SARS-CoV-2 reactive T-cells

Recent publications [23], [28], [29], [30], [31], [32] identifying epitopes on CD4 and CD8 T-cells against SARS-CoV-2 in convalescent blood after natural infection represent a key step toward understanding the role of adaptive T-cell responses in COVID-19 protective immunity. Sette and Crotty’s laboratories [23] reported that CD4 T-cell responses are less dominated by spike protein epitopes than in previous coronavirus infections. Spike accounted for 27% of total responsive CD4 T-cells, with membrane (M) and nucleocapsid (N) proteins accounting for 27% and 11%, respectively. In comparison, spike protein accounted for ~2/3 of reactive CD4 T-cells after previous coronavirus infections in humans [23], with one study [33] reporting no M or N CD4 response in recovered SARS-CoV-1 patients.

The results were even more striking for CD8 T-cells. Spike-reactive CD8 T-cells comprised only 26% and M 22% of the total CD8 responsive cells, while nsp6, ORF3a, and N comprised ~50%. This is very different from prior coronavirus infections, where spike generally contributed ~50% and N comprised 36%, although Zhao et al [11] showed broad responses to spike, N and M in MERS survivors.

Other studies have confirmed the breadth of CD4 and CD8 T-cell responses in COVID-19 convalescent patients. M- and N-reactive cells [10], [30], [31] are equal to or more prevalent than spike-reactive CD8 and CD4 T-cells.

These findings carry a potentially important message for SARS-CoV-2 vaccines. Most current vaccine candidates are focusing on spike protein as the immunizing antigen, but natural infection induces broad epitope coverage in T-cells. It will be essential to understand the relation between breadth, durability and quality of T-cell responses and resulting protective immunity with SARS-CoV-2 vaccines and natural infection.

## 4 T-cell and antibody responses in relation to COVID-19 disease severity

T-cell and antibody responses correlate with severity of COVID-19 clinical disease. Recovered patients with mild disease had more prevalent M- and N- than spike-responsive CD8 T-cells and more CD8 than CD4 virus-specific T-cells [30], compared to patients with severe disease. Patients with milder disease [32] also show greater clonal expansion and less active proliferation in CD8 T-cells in bronchial fluid as well as lower serum cytokine levels, compared to patients with severe disease. COVID-19 patients with serious illness or who died had higher expression of the programmed cell death 1 receptor in T-cells, consistent with T-cell exhaustion [14]. These findings suggest that the adaptive CD8 T-cell immune response in general and broad T-cell specificity in particular confer protective rather than pathologic effects. Peng et al. [30] concluded that “the identification of T cell specificity and functionality associated with milder disease highlights the potential importance of including non-spike proteins within future COVID-19 vaccine design.”

## 5 Benchmarks for T-cell immune response to viral vaccines: durability of virus-reactive T-cells as a metric of quality and longevity of immune protection

We are fortunate to have in Yellow Fever (YF) and smallpox vaccines excellent benchmarks for primary immune response to viral vaccination. These vaccines induce remarkably effective and long-lived immune protection and share common features for cellular immunity: generation of CD8 T-cells with broad specificity, high magnitude, polyfunctionality, high proliferative potential and long-term persistence [1]. This CD8 T-cell response pattern provides us with criteria for evaluating long-term, “high quality” protective immunity in vaccine trials or after natural infection.

In this context, recent data on the durability and cross-reactivity of SARS-CoV-2 responsive T-cells in exposed and unexposed subjects may have important clinical implications. LeBert et al. [28] reported remarkable results about T-cells that react to the coronavirus structural protein N in a Singapore population. Prevalence of reactive T-cells to SARS-CoV-2 N in was 100% in COVID-19 recovered patients (36/36 patients) and included coverage of multiple regions of the N protein. Moreover, 23/23 patients studied 17 years after recovery from SARS-CoV-1 infection still had reactive T-cells to SARS-CoV-1 N. Importantly, these cells also reacted to SARS-CoV-2 N. Finally, ~50% (19/37) of subjects who were never clinically exposed to either SARS-CoV-1 or −2 infection exhibited SARS-CoV-2 N-reactive T-cells.

These results in unexposed humans for N-reactive T-cells are supported by findings for spike-reactive T-cells [29]. T-cells that react to SARS-CoV-2 spike protein were present in 34% of seronegative, clinically unexposed healthy controls in a German cohort. COVID-19 recovered patients in this population showed 83% prevalence of SARS-CoV-2 spike-reactive T-cells at higher levels than in unexposed subjects. An intriguing finding was that the COVID-19 recovered patients’ T-cells covered epitopes broadly spaced across the spike protein, whereas the epitopes covered in control subjects were mostly against the C-terminal region, which has greater homology to spike proteins in betacoronviruses that cause the common cold. Prevalence of T-cells that are reactive to SARS-CoV-2 proteins has also been reported in unexposed subjects in studies from the United States [23], Sweden [10] and the Netherlands [24], although not in studies from Wuhan China [34] or the United Kingdom [35].

Taken together, these findings tell an important and potentially promising story. After SARS-CoV-1 or −2 infection, T-cells reactive to coronavirus proteins are universally observed, persist for many years and exhibit cross-reactivity between the two viruses. Moreover, SARS-CoV-2 reactive T-cells may often be induced by mild betacoronavirus infections that cause common colds. The finding of SARS-CoV-2-responding T-cells in unexposed subjects has now been reported in several geographic locations and, if it holds up to further study, might explain some of the variability of clinical outcomes for COVID-19. This observation also suggests potentially testable protective strategies for COVID-19 prevention through exposure to betacoronavirus common colds (the original vaccine of Jenner against smallpox, of course, was induced by intentional natural infection with a zoonotic virus that caused mild symptoms in humans).

**Fig. 1:**
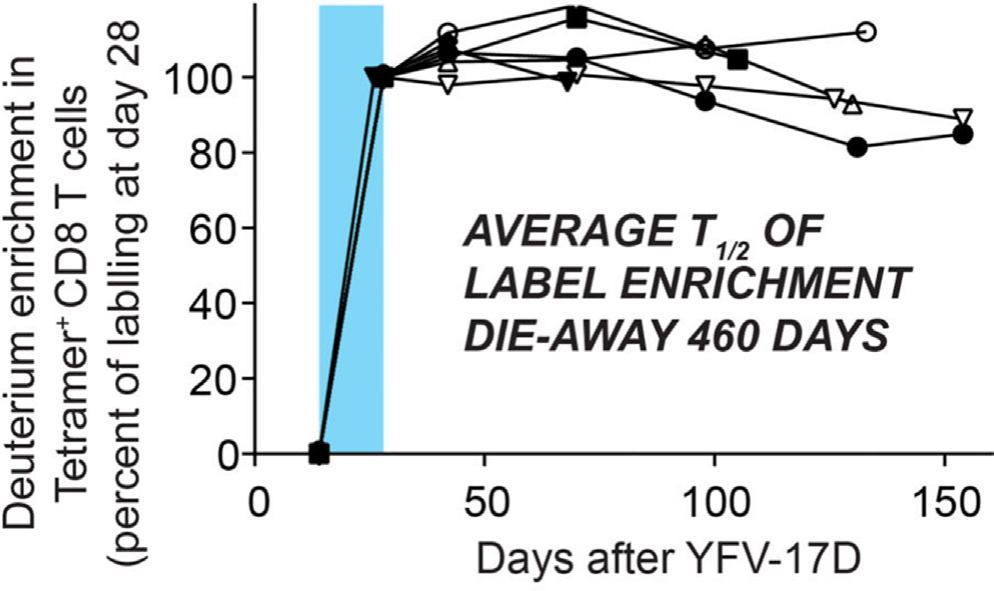
Long lifespan and mitotic quiescence of YFV-specific CD8 T-cells after vaccination (heavy water labeling shown in blue) (from [36]). (For interpretation of the references to colour in this figure legend, the reader is referred to the web version of this article.)

The apparent durability of virus-specific T-cells against SARS-CoV-1 [8], [9] after natural infection is also a central feature of highly effective viral vaccines - in particular, YF and smallpox vaccinations [1], [36]. To understand the basis of long-lived, high quality protective immunity following viral vaccination, we [36] recently characterized the lifespan and differentiation of YF virus (YFV)-specific CD8 T-cells (Fig. 1) after vaccination and *in vivo* labeling [37]. Long inter-mitotic interval was an early feature of YFV-specific CD8 T-cells generated. Long lifespan allowed differentiation from effector cells that proliferated during the initial viral exposure to a unique, stem-like memory T-cell population. These mitotically quiescent YFV-reactive cells maintained an epigenetic fingerprint of their effector history with open chromatin profiles at effector genes even a decade after vaccination. Indeed, these long-lived T-cells combine features of naïve and effector cells - surface markers (CD45RA and CCR7) and lack of granzyme B expression (naïve cell characteristics), rapid proliferation in response to viral antigens or cytokines (effector), and gene expression patterns distinct from either type. Importantly, long life-span of virus-specific T-cells was apparent within 1–4 months after vaccination by monitoring the slow die-away of labeled YFV-reactive T-cells (Fig. 1). It may therefore be possible to characterize very early after vaccination the quality and the durability of induced T-cell immune protection. As Flaxman and Ewer suggested [38], vaccine developers could use T-cell measurement methods “to evaluate vaccine-specific T-cells”.

## 6 Implications of T-cell findings in coronavirus infections for vaccine candidates

**Fig. 2:**
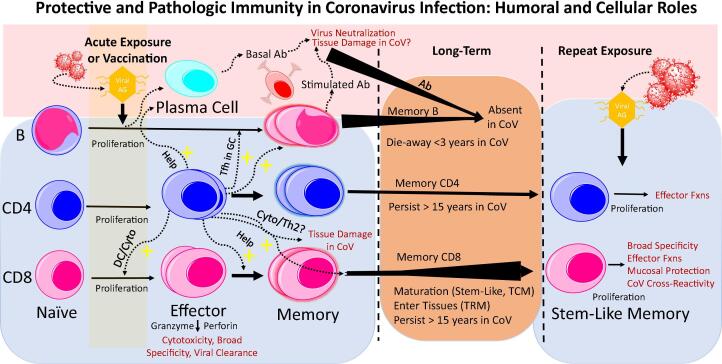
*Protective and Pathologic Immunity in Coronavirus Infection: Humoral and Cellular Roles*. Long-term survival and rapid proliferation with effector function on re-exposure are benchmarks of T-cells in highly effective infection- or vaccine-induced protective immunity against viral infections. Long-lifespan and maturation of CD8 T-cells is key for both quality and durability of immunity. In CoV infections, T-cells exhibit these features but antibodies and memory B-cells have not been durable. CD4 T-cells play critical helper roles for both CD8 T-cells and B-cells. Antibodies (by ADE or macrophage activation) and CD4 T-cells (by excessive cytokine production or Th2 eosinophilic immune damage) are concerns for potential contribution to tissue pathology in CoV infection. CD4 T-cells and tissue cytokines have shown a Th1 pattern in SARS-CoV-2. T-cells in CoV infections appear to have long lifespan and in both SARS-CoV-1 and -2 patients there cross-reactivity for betacoronavirus proteins. Abbreviations: CoV, coronavirus; Ab, antibodies; Ag, antigen; Cyto, cytokines; DC, dendritic cells; TCM, central memory T-cells; TRM, resident memory T-cells; T_fh_, follicle helper T-cells; GC, germinal center; ADE, antibody-dependent enhancement; Fxns, functions; +, stimulatory effect.

T cells interact with humoral immunity in several ways that can influence both protective immunity and tissue pathology. Knowledge is advancing on how this plays out for natural coronavirus infections (Fig. 2). Protective natural immunity to coronavirus infections, including SARS-CoV-2, provides criteria for vaccine evaluation. In particular, CD8 T-cells with broad specificity (not just to spike protein) and long persistence, more than a robust antibody response alone, may be a signature of successful protective immunity against SARS-CoV-2 and SARS-CoV-1 infections.

A key early question for any candidate vaccine for COVID-19 will therefore be whether it induces durable, high quality T-cell protective immunity. A theoretical advantage of mRNA vaccines, for example, is that antigens are synthesized in the cell cytosol where they can be processed and bound to MHC-class I molecules on the cell surface for recognition by CD8 T-cells. But it is not known whether the absence of SARS-CoV-2 antigens such as M or N will mimic the functionality of T-cell responses from natural infection or what the durability of induced T-cells will be. The same uncertainties hold true for any spike protein based COVID-19 vaccine.

Preliminary reports on humoral and T-cell responses have been published for three SARS-CoV-2 vaccine candidates in humans [34], [35], [39] and for candidates in non-human primates [40], [41]. Two of the human vaccines involved adenovirus-vector delivery of spike protein and one was a spike-based mRNA vaccine. All reported induction of anti-spike antibodies and spike-reactive T-cells over a 1–2 month follow-up period, but deeper characterization of T-cell responses, including specificity, magnitude, polyfunctionality, proliferative potential and long-term persistence, have not been reported and remain key unknowns. The reports in non-human primates involved an adenovirus vector-based [40] and a DNA [41] vaccine expressing the SARS-CoV-2 spike protein. Both induced anti-spike antibodies and spike-reactive T-cells after 2–4 weeks and protection against viral challenge at 6 weeks. Durability of the antibody response or durability and quality of the T-cell response were not assessed.

Grifoni et al. [23] wrote about natural infection that “knowledge of the T-cell responses to COVID-19 can guide selection of appropriate immunological endpoints” for SARS-CoV2 vaccine trials. In contrast to the H1N1 vaccine, which was developed and licensed 5 months after the first U.S. case was identified in the H1N1 flu outbreak in 2009, the situation is very different for COVID-19 [42]. There was extensive experience with influenza vaccine technology and vaccines made using well established platforms were licensed under the rules for a strain change. There has never been a coronavirus vaccine or an mRNA vaccine, and until there is a definitive Phase 3 efficacy trial we will have to depend on laboratory measurements and their correlations with clinical outcomes.

It would be a public health and “trust-in-medicine” nightmare with potential repercussions for years - including a boost to anti-vaccine forces - if immune protection wears off or antibody-dependant enhancement develops and we face recurrent threats from COVID-19 among the immunized. Data correlating clinical outcomes with laboratory markers of cell-mediated immunity, not only with antibody responses, after vaccination or natural infection with SARS-CoV-2 or other betacoronviruses may prove critically valuable, particularly if protective immunity fades or new patterns of disease emerge.

## Declaration of Competing Interest

The authors declare that they have no known competing financial interests or personal relationships that could have appeared to influence the work reported in this paper.
